# Supplementary figures and images for: Potential of Aqueous Humor as a Liquid Biopsy for Uveal Melanoma
Source: Int J Mol Sci. 2022 Jun 2;23(11):6226. doi: 10.3390/ijms23116226 (PMC9181140; doi:10.3390/ijms23116226)

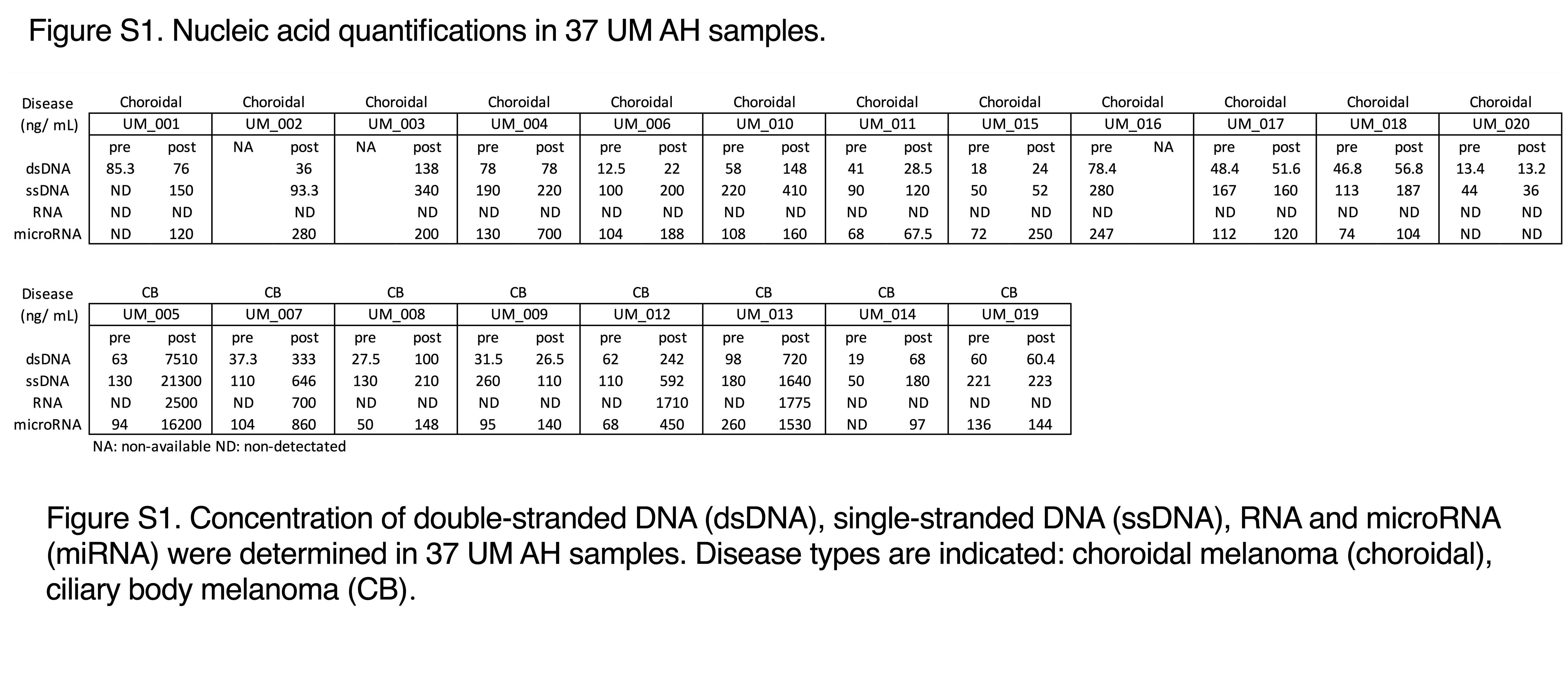

Supplement: Supplementary file 1 [file ijms-23-06226-s001.zip › FigureS1.tiff]

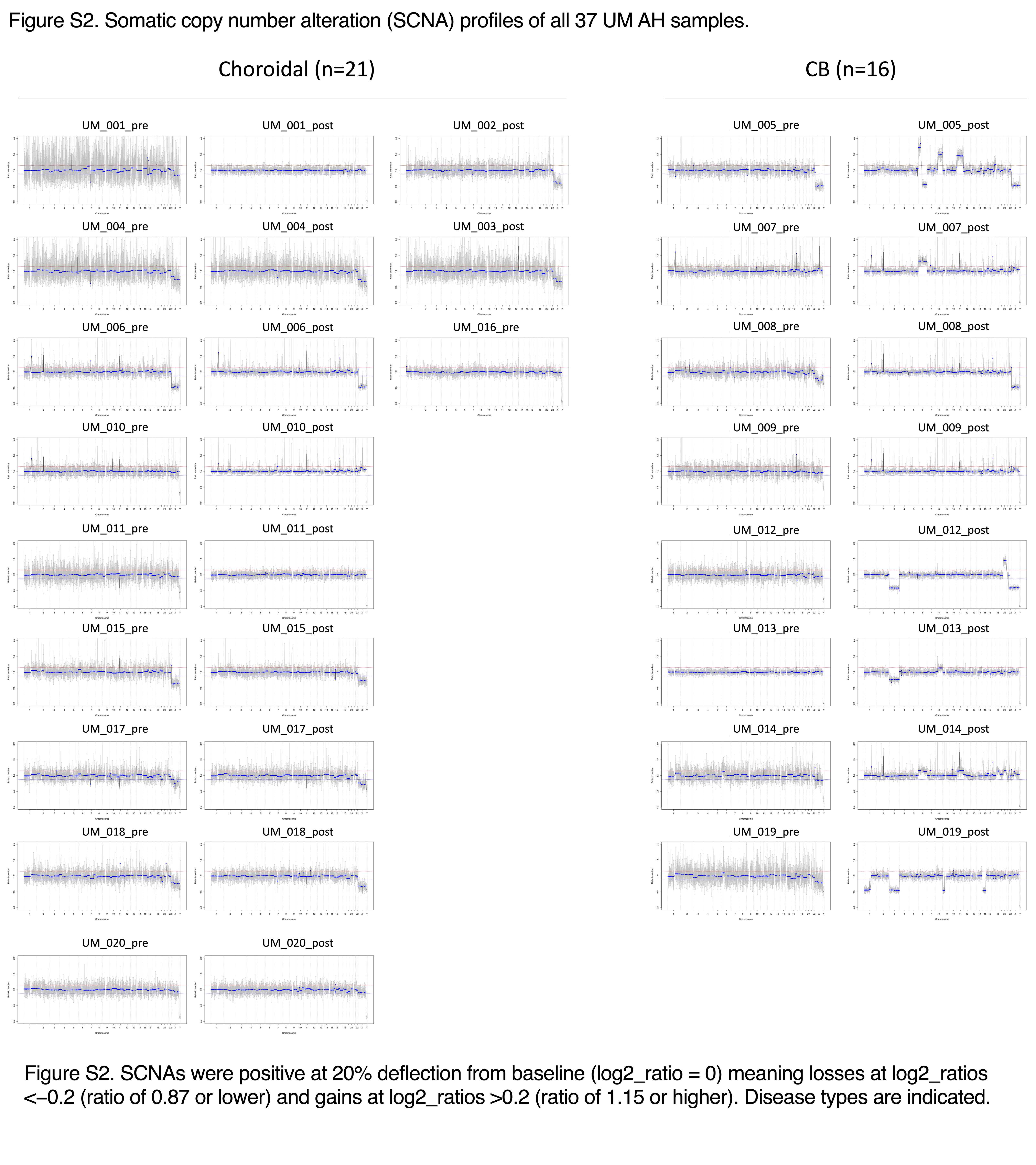

Supplement: Supplementary file 1 [file ijms-23-06226-s001.zip › FigureS2.tiff]

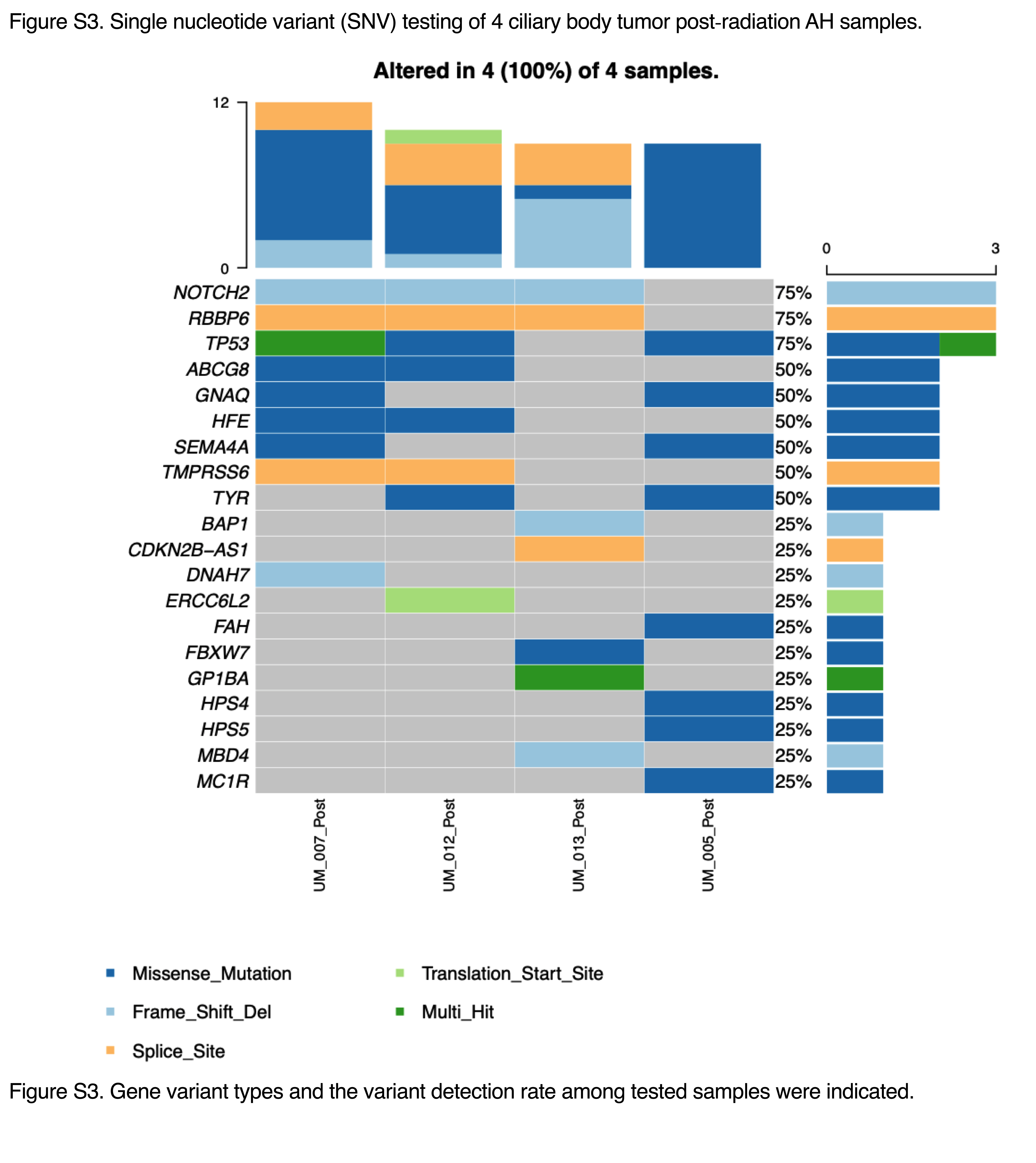

Supplement: Supplementary file 1 [file ijms-23-06226-s001.zip › FigureS3.tiff]
